# Analysis of the spatio-temporal evolution of iron ore trade from a geopolitical perspective: A complex network model

**DOI:** 10.1371/journal.pone.0345177

**Published:** 2026-03-24

**Authors:** Shengzhong Huang, Chenrui Cai, Bo Fu

**Affiliations:** 1 School of Management, China University of Mining and Technology -Beijing, Beijing, China; 2 Research Institute of Decision-making Science and Big Data, China University of Mining and Technology -Beijing, Beijing, China; Shanghai Jiao Tong University, CHINA

## Abstract

Iron ore is the primary raw material for steel production, and the steel industry is a fundamental pillar of the national economy. Given the increasing strategic importance of iron ore, this paper introduces a complex network model to analyze the evolution characteristics of the global iron ore trade pattern from 2000 to 2023, aiming to assess the trade situation of iron ore. In addition, relevant policies have been formulated to ensure the stable supply of iron ore. The study shows that the Global Iron Ore Trade Network (GIOTN) is active and efficient. However, the iron ore trade is highly imbalanced and heavily influenced by geopolitical factors. Furthermore, under the impact of geopolitics, there is a gradual shift from resource dominance to capital dominance in the global iron ore trade. In terms of trade competition, relations between Europe and Brazil seem to be more intense, and the competitive landscape of iron ore trade in southern Africa is becoming increasingly prominent. Meanwhile, the global iron ore trade is becoming more regionalized due to geopolitical influences, with trade communities trending towards smaller and more balanced states. Emerging economies such as China, South Africa, and India are playing an increasingly important role in the global iron ore trade.

## 1. Introduction

Steel, as the cornerstone of modern industry, is widely used in critical sectors such as construction, transportation, energy, and manufacturing [1,2]. As the primary raw material for steel production [3], iron ore is not only a key resource supporting global infrastructure development and industrial growth but also holds significant strategic importance in the global economic landscape [4,5]. According to statistics from the World Steel Association [6], global iron ore production in 2022 was approximately 2.4557 billion tonnes, but its reserves are highly unevenly distributed, primarily concentrated in a few countries such as Brazil and Australia [7]. China, as the world’s largest importer of iron ore, has long had an import dependency rate exceeding 75% [8,9], making the international iron ore market highly sensitive to geopolitical events, climate disasters, and policy shocks [10]. Additionally, the diversity and complexity of trade participants further exacerbate market uncertainty [11]. Against the backdrop of an increasingly complex national geopolitical environment, how does the structure of the global iron ore trade network evolve over time? How do geopolitical factors exert long-term, systemic shaping effects within it? These are critical questions that urgently require resolution. Therefore, systematically studying the structural characteristics of the iron ore trade network and its dynamic evolution under geopolitical influences holds significant practical implications for understanding global resource security and enhancing supply chain resilience.

Existing research primarily focuses on the market supply and demand dynamics, pricing mechanisms, and risk sensitivity of iron ore. Cheng et al. [12] emphasize the need to consider both economic and environmental factors influencing iron ore in an uncertain market environment. Chen et al. [13] confirmed that fluctuations in iron ore prices are driven by demand factors and align with the supercycle theory. Pan et al. [14] proposed an advanced METL model to predict iron ore prices, addressing the uncertainty of iron ore price fluctuations. Bhattacharya et al. [15] pointed out that exchange rate fluctuations are a key determinant of iron ore export revenues. Rabab et al. [16] employed dynamic material flow analysis to reveal the key characteristics of Pakistan’s iron metabolism during the 2005−2020 period. In recent years, some studies have begun to explore the impact of geopolitics on resource trade patterns. For example, Khurshid et al. [17] assessed the impact of geopolitical risk (GPR) on natural resources (NR) using monthly and daily data. Through causal analysis, the researchers identified the Russia-Ukraine conflict as a key driver behind the surge in natural resource (NR) prices. This causal link was clearly demonstrated by comparing actual price trends with counterfactual projections simulating a non-conflict scenario. Moreover, the study revealed that the price impacts of geopolitical conflicts are not uniform across commodities, as predictions under conflict conditions diverged variably from those in peaceful scenarios depending on the type of resource. The study results confirm that NR may not be affected if no intervention is made. Yang et al. [18] explored the positive correlation between rare earth elements and the clean energy market under the backdrop of the COVID-19 pandemic and the Russia-Ukraine conflict. Geopolitical risks are an important factor driving changes in energy prices [19], and the Russia-Ukraine conflict can increase gasoline prices by influencing international crude oil prices [20]. Ke et al. [21] noted that the Russia-Ukraine conflict has altered the structure of LNG transportation networks and weakened their resilience. Gopinath et al. [22] estimated using a gravity model that since the outbreak of the war in Ukraine, trade and portfolio flows between countries in the same group that are geographically distant from each other have declined significantly compared to flows between countries in the same group.These findings reflect that geopolitics has become an important contextual variable in resource trade changes. However, while these studies reveal the connection between geopolitics and resource trade, they primarily focus on individual event shocks, price fluctuations, or localized path changes, and have not yet explored, from the perspective of systemic structural evolution, how geopolitical landscapes are embedded and reshape international iron ore trade networks over the long term.

To address the limitations of traditional research in structural identification and system modeling, complex network theory has increasingly been applied to global resource trade studies [23]. Cai et al. [24] constructed a trade network map between countries and utilized metrics such as node centrality, clustering coefficient, and modularity to identify key nodes and vulnerable paths within the trade network. Zheng et al. [25] found that the kaolin trade network is becoming increasingly tight and efficient. In research combining resource security and geopolitics, Wang et al. [26] constructed a structural evolution map of global scrap copper trade from 1988 to 2017, showing that global scrap copper trade has been reshaped by the interaction between geopolitical relations and geoeconomics. Baranowski et al. [27] analyzes Russia’s pivotal role in controlling strategic minerals during its invasion of Ukraine from an adaptive resource geopolitics perspective. Wang et al. [28] ssesses geopolitically driven supply risks for critical metal minerals. These studies demonstrate that geopolitical landscapes indeed exert significant shaping power over the structure of global resource networks. However, most studies focus on specific geopolitical events or mineral types, with analytical scales limited to individual regions or short-term conflicts. There is a lack of analytical frameworks that systematically reveal how geopolitics is deeply embedded in resource trade structures and drives their long-term spatiotemporal evolution from a global network perspective. Particularly regarding iron ore, a strategic resource, there remains a shortage of comprehensive studies that integrate geopolitical contexts, structural characteristics, and evolutionary trends.

Although existing research has provided a theoretical foundation for understanding the international iron ore trade network, there are still some shortcomings: first, current research often lacks a systematic examination of the dynamic evolution of trade networks over time and across geopolitical landscapes; second, the application of complex network analysis is largely limited to static structures or risk propagation modeling, lacking an integrated perspective on network structure and geopolitics. Therefore, the “Global Iron Ore Trade Network” (GIOTN) is defined as a weighted network composed of countries around the world as nodes and iron ore trade volumes as directed edges, used to describe the structural characteristics and evolutionary patterns of cross-border iron ore resource flows on a global scale. The focus of the research is to systematically study the spatiotemporal evolution logic of GIOTN from a geopolitical perspective using complex network methods. The main contributions of this research include: (1) A systematic analysis of GIOTN structural changes from a spatio-temporal perspective, revealing the evolution characteristics of trade flows across different periods and regions. (2) Constructing a GIOTN model to identify core trading nations, dominant pathways, and community structures, providing tools to understand the stability and potential risks of international trade. (3) Analyzing the impact of geopolitical landscape changes on iron ore trade structures from a geopolitical perspective, proposing policy recommendations to enhance iron ore resource supply security, and providing theoretical references for constructing a stable and efficient global iron ore resource governance system.

The remainder of this paper is structured as follows: Section 2 introduces the data sources and modeling methods. Section 3 provides an overview of the overall situation of iron ore trade, the spatiotemporal trade patterns of core countries, and the division of network communities. Section 4 presents the research results and discusses them. Section 5 summarizes the findings and proposes policy recommendations.

## 2. Methods and data

**Fig 1:**
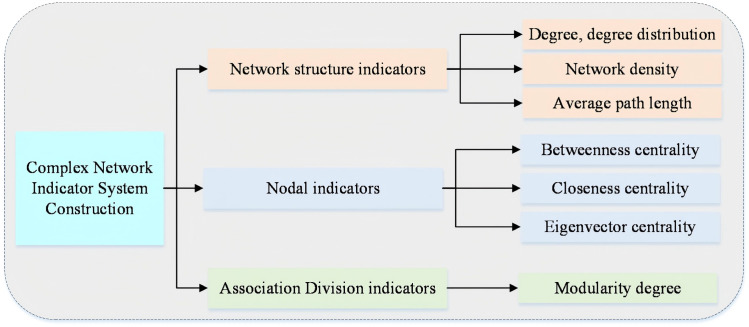
GIOTN indicator system construction process GIOTN.

**Fig 2:**
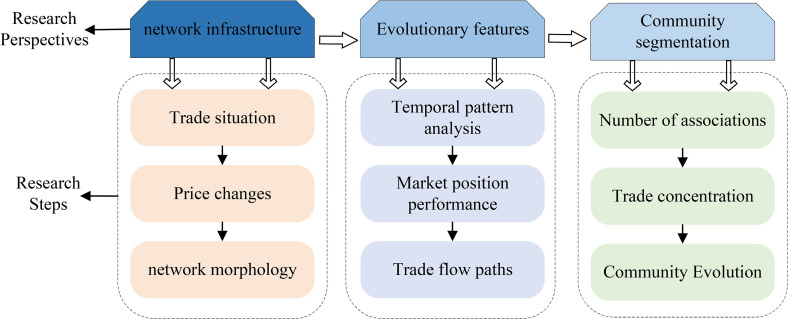
Research framework.

This paper constructs the Global Iron Ore Trade Network (GIOTN) and explores the trade patterns and evolution trends of international iron ore trade by analyzing the network’s topological parameters, node characteristics, and community structure. The construction process of the GIOTN indicator system is shown in (Fig 1), and the research framework is shown in (Fig 2). Network structure, node characteristic analysis, and community detection are important core components of network system research. Network structure is described through topological attributes, with common metrics including average path length, clustering coefficient, and network diameter, which are used to measure network connectivity and compactness. Node feature analysis evaluates the importance of each node in the network, with common metrics including degree, betweenness centrality, closeness centrality, and eigenvector centrality. These metrics help identify key nodes and information transmission hubs within the network. Community detection groups closely connected nodes to reveal the modular structure within a network, thereby identifying internal communities or functional groups. By integrating these metrics, one can gain a comprehensive understanding of the network’s overall characteristics, the role of nodes within the network, and the network’s hierarchical structure, providing valuable insights for optimizing network functionality.

This paper systematically characterizes GIOTN from three dimensions: first, based on the perspective of network infrastructure, it conducts research through three levels of trade situation analysis, price fluctuation characteristics, and network structure; second, from the dimension of evolutionary characteristics, it conducts dynamic investigations using methods such as time pattern analysis, market position assessment, and trade flow path tracking; finally, it focuses on community segmentation characteristics and presents them from multiple angles through correlation quantity statistics, trade concentration measurement, and community evolution pattern analysis.

### 2.1 Complex network construction

Considering the directionality and non-equivalence of trade relationships, a weighted directed GIOTN was constructed based on bilateral iron ore trade data. The entire trade network is represented by the set G=(V,E). Countries involved in iron ore trade are defined as nodes V; $V\left(v_{\text{1}},v_{\text{2}},v_{\text{3}}\cdots v_{n}\right)$ represents a network node, indicating a country participating in international iron ore trade. Trade relationships between countries are defined as edges E, with the direction of iron ore trade flow as the direction of the edges. $E_{ij}$ represents a network edge, indicating the trade relationship between exporting country i and importing country j. The trade volume is defined as the weight of the edge $\omega_{ij}$, representing the volume of iron ore trade exported from i to j. A directed weighted network of global iron ore trade was constructed for each year, resulting in 24 complex networks. The network is represented using an adjacency matrix A(t), as shown in the formula.


$$\begin{array}{c} A\left(t\right)\left[\begin{array}{ccc} @ccc@w_{1,1}\left(t\right) & \cdots & w_{1,j}\left(t\right)\\ \vdots & \ddots & \vdots \\ w_{i,1}\left(t\right) & \cdots & w_{i,j}\left(t\right) \end{array}\right] \end{array}$$ (1)

The indicators for the iron ore trade network are based on the papers [29–32], with detailed discussions provided in (S2 Appendix) Formulas 1–11. S2 Appendix Formula 1–5 describes degree and degree distribution, Formula 6 describes network density, and Formula 7 describes average path length [33]. These are descriptive indicators for network structure. Formula 8 characterizes intermediate centrality, Formula 9 characterizes proximity centrality, and Formula 10 characterizes eigenvector centrality [34]. These formulas are descriptive metrics for node importance. Formula 11 describes modularity, characterizing network community partitioning.

### 2.2 Data

This study selects bilateral iron ore international trade data between 2000 and 2023 from 195 countries and regions, with data sourced from the United Nations Commodity Trade Statistics Database (UN Comtrade). Iron ore is identified by HS code 260111, corresponding to the commodity “Iron ores and concentrates; non-agglomerated.” The trade data is measured in terms of physical quantity (unit: kilograms) and monetary value (unit: US dollars). Each trade record corresponds to an importing and exporting country, with all countries being reporters of the UN trade data. Since customs inspections are stricter on the import side, import data is generally more accurate [35]. This study excludes non-significant trade relationships (i.e., trade volumes below 100 kilograms) to highlight major trade relationships.

## 3. Analysis of results

### 3.1 General overview of the global iron ore trading network

This section investigates the GIOTN from 2000 to 2023, focusing on its developmental trajectory and core patterns along two dimensions, trade volume dynamics and network structural characteristics. First, for trade scale and price fluctuations, the study analyses the quantity, total value and volatility of global iron ore trade and the underlying drivers, as discussed further in Section 3.1.1. Second, for network structure, topological properties of the GIOTN, including number of nodes, edges, average degree, average path length and network density, are quantified and examined together with the power law distribution of scale free networks to reveal dynamic evolutionary patterns. Preliminary results show that the number of nodes increased from 103 in 2000 to a peak of 141 in 2011, falling to 123 in 2023; the average degree reached a maximum of 11.197 in 2021, indicating notably stronger trade linkages; the average path length decreased from 2.761 in 2003 to 2.266 in 2023, reflecting improved connectivity and trade efficiency; network density peaked at 0.044 in 2021, suggesting closer ties among trading economies. Annual variation trends are analysed further in Section 3.1.2.

Integrating findings from both dimensions, this study establishes a foundation for investigating the GIOTN’s evolutionary mechanisms. It captures both the dynamic expansion and contraction of trade scale and the complexity and heterogeneity of network structure, thereby constructing a comprehensive, multi‑level understanding of the global iron ore trade network.

#### 3.1.1 Analysis of global iron ore trade volume and its price change.

**Fig 3:**
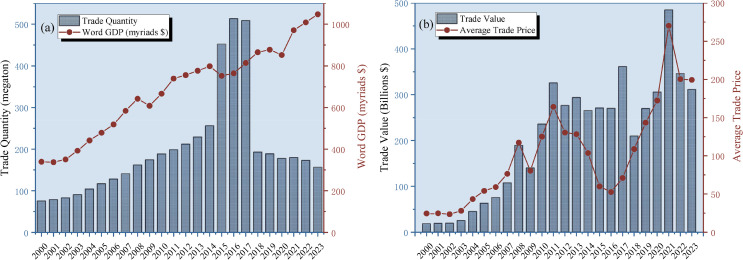
Global Iron Ore Trade Volume and Trade Value from 2000 to 2023. (Note: The average trade price is calculated by dividing the trade value of iron ore by the trade volume, The prices here correspond to the HS260111 iron ore commodity price above.).

Fig 3 presents the global iron ore trade volume and total trade value from 2000 to 2023, together with their relationships to global GDP and average trade price. Fig 3(a) shows that global iron ore trade volume generally increased over the period, with particularly strong growth from 2000 to 2016, rising from 75.5 million metric tons in 2000 to 513.5 million metric tons in 2016. This trend is closely associated with large scale infrastructure development and accelerated urbanization in emerging economies, such as China. During this period, the rapid growth in global demand for steel directly drove the expansion of iron ore trade. After 2014, despite continued growth in global GDP, the volume of global iron ore trade experienced a temporary decline from 2017 to 2018, marking an important turning point in the evolution of the global iron ore market. The main reasons include China’s launch of supply-side structural reforms, the implementation of environmental protection production restrictions, and frequent rainfall and safety accidents in Brazilian mining areas, which caused iron ore demand to slow down temporarily. Fig 3(b) reveals the fluctuations in the total value of iron ore trade and the average trade price. After the 2008 global financial crisis, although trade volume recovered rapidly, the total value of trade declined. The sharp price surge in 2010–2011 reflected heightened market expectations of supply-demand mismatches. The significant price decline around 2015 was linked to China’s steel industry overcapacity and the conclusion of global mining companies’ expansion cycles. The extreme high prices in 2021 were primarily driven by COVID-19-induced global supply chain disruptions, coupled with China’s robust post-pandemic economic recovery, which sparked a surge in iron ore demand. In contrast, trade prices and total value declined significantly in 2022–2023, due to a combination of factors including slowing global economic growth, the Federal Reserve’s interest rate hikes suppressing commodity prices, and adjustments in China’s real estate market, which led to a cooling of the iron ore market.

In summary, the differing trends between Fig 3(a) and Fig 3(b) at different stages reflect that trade volumes are primarily influenced by long-term industrial structure and policy adjustments, while trade prices are more sensitive to short-term supply-demand changes and global economic events. Together, these factors highlight iron ore’s critical role as a strategic commodity within the global economic cycle.

#### 3.1.2 Analysis of global iron ore trading network structure.

**Fig 4:**
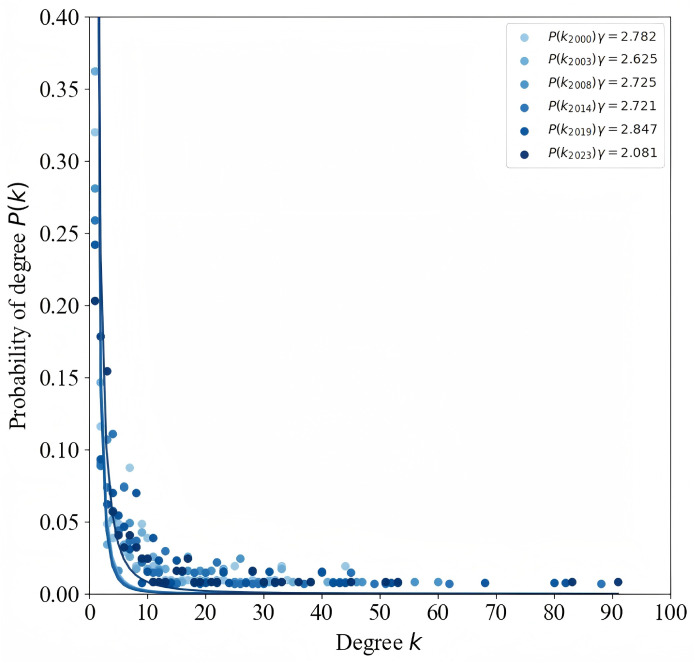
Degree distribution of GIOTN at key time points. (Note: Different colored dots represent data for different years. The horizontal axis represents the node degree value K, and the vertical axis represents the corresponding probability ρ(K). The degree distribution is calculated based on the probability of randomly selecting a node in the network whose degree is exactly K.).

As shown in (Table 1), the average number of nodes in the GIOTN from 2000 to 2023 is 125, accounting for 56% of all countries and regions globally. This indicates that more than half of the world’s countries and regions participate in iron ore trade. Before 2011, the number of nodes showed a fluctuating upward trend, increasing from 103 to 141, indicating that more economies were joining the global iron ore trade. At the same time, the number of edges also increased, peaking in 2021, reflecting the increasing complexity of global iron ore trade relationships and the network structure. Additionally, the average degree reached its peak in 2021, signifying the highest level of overall trade prosperity at that time. The average path length decreased from 2.761 in 2003 to 2.266 in 2023, indicating that the trade distance between economies in the iron ore trade network is shortening and the network’s connectivity is improving, enhancing overall trade efficiency. The average path length reflects the trade efficiency between economies. In 2021, the network density reached 0.044, indicating stronger ties between economies participating in trade. To further evaluate the characteristics of the global iron ore trade network, a power-law function was used to fit the degree distribution of the trade network. The fitting results suggest that the GIOTN approximates a scale-free network, as shown in (Fig 4). This means that the importance of nodes in the GIOTN is highly heterogeneous, with significant differences in the trade status and roles of various economies within the trade network.

**Table 1:** Changes of topology parameters of GIOTN from 2000 to 2023.

Year	Nodes	Edges	Average degree	Average path length	Density
2000	103	404	7.845	1.804	0.038
2001	102	423	8.294	1.487	0.041
2002	115	415	7.217	1.798	0.032
2003	116	422	7.276	2.761	0.032
2004	114	454	7.965	2.652	0.035
2005	115	498	8.661	2.568	0.038
2006	123	506	8.228	2.581	0.034
2007	130	536	8.246	2.639	0.032
2008	121	552	9.124	2.555	0.038
2009	123	503	8.179	2.561	0.034
2010	130	554	8.523	2.692	0.033
2011	141	626	8.879	2.604	0.032
2012	127	665	10.472	2.516	0.042
2013	138	638	9.246	2.512	0.034
2014	135	635	9.407	2.657	0.035
2015	132	630	9.545	2.407	0.036
2016	130	619	9.523	2.413	0.037
2017	137	662	9.664	2.466	0.036
2018	132	673	10.197	2.520	0.039
2019	128	705	11.016	2.436	0.043
2020	126	612	9.714	2.410	0.039
2021	127	711	11.197	2.467	0.044
2022	132	697	10.561	2.268	0.040
2023	123	552	8.976	2.266	0.037

### 3.2 Analysis of spatial and temporal patterns of core trading countries

**Fig 5:**
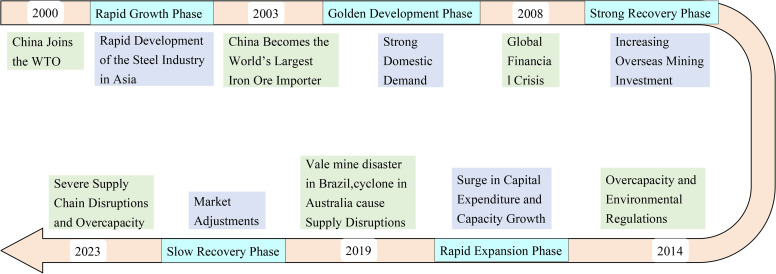
Timeline of key trade milestones in the GIOTN.

Supply and demand analysis is a fundamental method in economics for analyzing market supply and demand. When supply and demand are in balance, the market is in equilibrium; conversely, when supply and demand are out of balance, the market is in disequilibrium. It is necessary to adjust the supply-demand relationship to restore balance and stability. Therefore, this study uses supply and demand as a starting point to divide the turning points in global iron ore supply and demand into different stages of trade evolution. As shown in (Fig 5), this study identifies six key time points and five stages. Among these, the rapid growth stage [36] occurred between 2000 and 2003, driven by the rapid development of the Asian steel industry; the period from 2003 to 2008, characterized by China’s strong domestic demand, represents the golden development phase [37,38]; the period from 2008 to 2014, marked by increased overseas mining investments, constitutes the strong recovery phase [39]; The surge in capital expenditure and capacity between 2014 and 2019 [40]; The period from 2019 to 2023 represents the market adjustment phase [41]; This provides a foundation for subsequent research on GIOTN. Based on the above analysis, subsequent examination of the global iron ore trade network from 2000 to 2023 will focus on the core critical time points identified earlier. Previous analyses have confirmed the scale-free nature of GIOTN. Therefore, focusing on core trading nations can help us better understand the evolving trends in global iron ore trade. This paper analyzes the market positions and trade routes of core trading nations from both temporal and spatial dimensions. From a temporal evolution perspective, the evolution of core trading nations’ market positions can be divided into three parts: first, absolute trade position, which refers to a nation’s overall share and influence in global iron ore trade. Second, key trade status: this emphasizes a country’s strategic importance in connecting different trade regions and facilitating trade flows. Third, dominant trade status: this highlights a country’s leadership role in shaping the global iron ore trade landscape. From a spatial evolution perspective, this paper primarily examines the changes in trade flows of core trading nations. Through these analyses, the study aims to reveal the spatio-temporal characteristics of the evolution of the global iron ore trade network.

#### 3.2.1 Market position analysis of core trading countries.

(1) Absolute trade position

**Fig 6:**
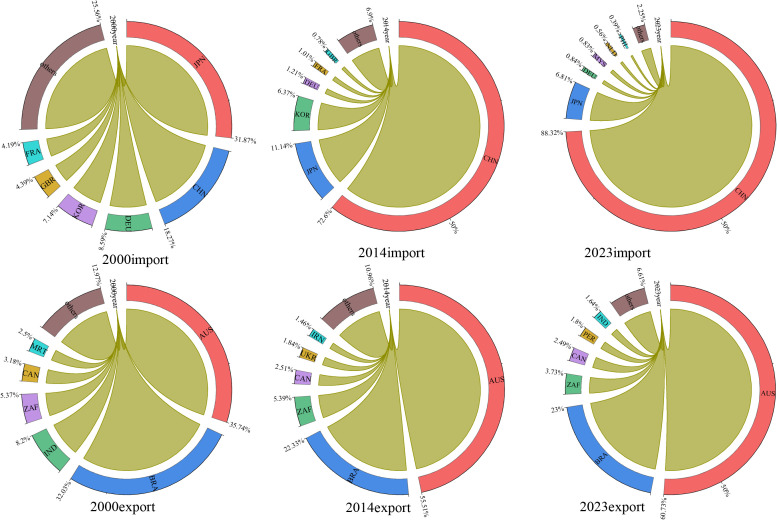
Changes in the share of iron ore trade export and import among major participating countries.

To examine the phased evolution of global iron ore trade shares, this study adopts the time‑node divisions from Fig 5 for the analysis of Fig 6. the global iron ore trade exhibits significant structural imbalances between supply and demand. Australia and Brazil are the core suppliers in the global iron ore trade, accounting for a combined 83.7% of global exports in 2023, forming a significant duopoly. This structure stems from differences in resource endowments and technological capabilities: Australia possesses abundant and high-quality iron ore resources, coupled with mining technology upgrades supported by international capital, resulting in its export share steadily increasing from 35.7% in 2000 to 60.7% in 2023; As shown in (S1 Appendix), while Brazil possesses vast iron ore reserves, its relatively backward technological capabilities have constrained its mining capacity, resulting in its export share declining from 32% to 23%. On the demand side, a structural shift dominated by China has emerged. In 2000, the global iron ore import market was dominated by six countries-Japan, China, Germany, South Korea, the United Kingdom, and France-which collectively accounted for 72.7% of total imports. Among these, Japan was the largest iron ore importer, accounting for 31.1%, while China accounted for only 17.8%. China’s import share has continued to grow annually due to its rapid industrialization and expansion of steel production capacity, surpassing Japan in 2003 with a 33.1% share and reaching 88.3% by 2023. Meanwhile, Japan’s share dropped to 6.8%. This trend, combined with the industrialization processes of Asian developing countries such as Vietnam and Malaysia, has driven annual growth in import volumes from emerging markets since 2019, leading to a core-periphery structure in the global iron ore trade network characterized by Australian ore concentration on the export side and Chinese dominance on the import side.

(2) Key trade position

**Fig 7:**
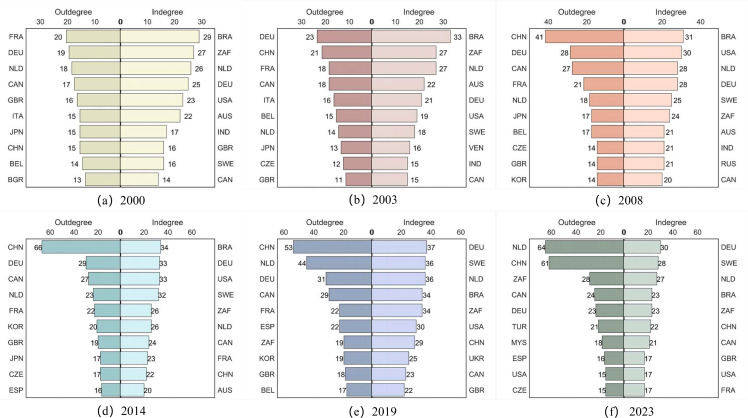
In-degree and out-degree of the top ten countries in GIOTN.

As shown in (Fig 7), the global iron ore trade landscape exhibits significant spatio-temporal differentiation. On the export side, the market is driven by resource endowments. From 2000 to 2003, Brazil, South Africa, and the Netherlands dominated the iron ore export market. However, from 2008 to 2014, the United States emerged as a major exporter due to the technological spillover effects of the shale revolution, while the positions of South Africa and the Netherlands declined. From 2019 to 2023, European industrialized countries such as Germany and Sweden have occupied the top three positions in the market, leveraging their mature industrial systems. (In subsequent references to ‘Europe’ or ‘the European Union,’ this article strictly distinguishes between the two concepts. Europe: Refers to the geographical continent of Europe, a category based on natural geography, which is traditionally divided into five regions: Northern Europe, Western Europe, Central Europe, Southern Europe, and Eastern Europe. The European Union (EU), located in the western part of the Eurasian continent, is a regional integration organisation comprising 27 European countries, combining both political and economic entities.) Meanwhile, Australia has maintained approximately 20 stable export partners, leveraging its high-quality iron ore resources and efficient logistics network to solidify its position as the world’s largest exporter. This resource-driven export structure underscores the foundational role of natural resource endowments in shaping trade patterns. The import side exhibits functional differentiation. China, as the world’s largest importer of iron ore, saw its import volume growth closely aligned with the expansion of steel production capacity; Germany demonstrated the resilience of European industrial demand through its stable top-three ranking; and the Netherlands surpassed China in 2023 with 64 import partners to become the largest trade network node. Meanwhile, the Netherlands’ number of export trade partners reached 27, ranking among the top three globally, highlighting its status as a Western European trade hub; however, its imports primarily serve the transshipment trade functions of the Port of Rotterdam. This trade hub-oriented import model underscores the economic logic of European coastal nations leveraging logistics value-added services to generate foreign exchange earnings. The asymmetry in the number of import and export partners, with the number of import trade partners exceeding that of exports, further validates the market concentration advantage of resource-exporting countries.

(3) Dominant trade position

**Fig 8:**
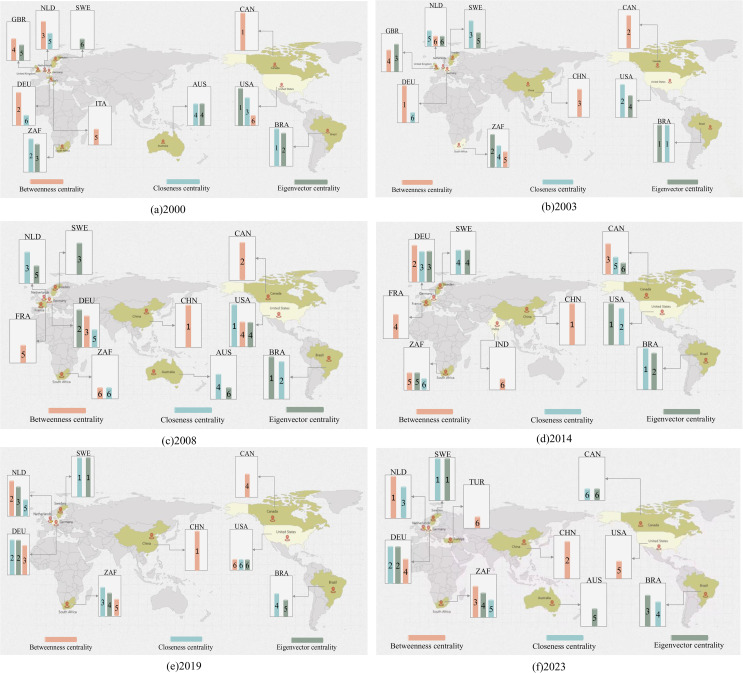
Top six countries ranked by betweenness centrality, closeness centrality, and eigenvector centrality. (Note: The numbers above the bar chart represent the ranking order of the network indicators corresponding to their respective colors. Map base data source: https://www.amcharts.com/javascript-maps/).

This section systematically assesses the evolution of countries’ control capabilities, autonomy, and influence in global iron ore trade from 2000 to 2023 using three network metrics: intermediate centrality, proximity centrality, and eigenvector centrality (Fig 8). In terms of control capabilities, trade dominance has shown a trend of shifting from traditional developed countries to emerging economies. While European and North American countries still dominate pricing power through their financial advantages, emerging markets such as China, South Africa, and India have significantly enhanced their bargaining positions through infrastructure upgrades, such as China’s increased steel production capacity driving a surge in iron ore demand. This has created a dual structure where developed countries set the rules and emerging economies share power. From the perspective of autonomy, iron ore-dominated countries like Australia, Brazil, and South Africa have relied on their abundant iron ore resources. Data from 2000 to 2023 shows that they have maintained trade surpluses by controlling resource extraction and exports, with their autonomy rooted in natural resource endowments. Capital-dominated countries like the United States and Germany, on the other hand, have leveraged their advanced financial systems and logistics infrastructure to control the flow of international trade through capital operations and infrastructure advantages. Both types of countries exhibit strong autonomy characteristics, but trends indicate that autonomy is shifting from the resource end to the capital end. As shown by the changes in closeness centrality rankings in Fig 8, in 2000 the leading countries were Brazil, South Africa, the United States, Australia, the Netherlands, and Germany, with the iron ore resource rich nations Brazil and South Africa occupying the top two positions, reflecting the dominant role of resource endowment in trade autonomy. By 2023, the ranking evolved to Sweden, Germany, the Netherlands, Brazil, South Africa, and Canada, with the capital driven countries Sweden, Germany, and the Netherlands rising to the top three, indicating the markedly strengthened effect of capital and logistics advantages in enhancing trade autonomy. While resource-rich countries can ensure domestic supply and export surplus products, capital-strong nations are gradually strengthening their control over resource trade through financial tools and infrastructure investments, forming a new power structure where resources provide the foundation and capital determines distribution. From the perspective of influence, the feature vector centrality index reveals the structural transformation of dominant forces in global iron ore trade. In 2000, resource-rich countries such as Brazil, South Africa, and the United States dominated the market, but after 2023, European developed coastal countries like Sweden, Germany, and South Africa rose to the forefront. This shift reflects how capital-intensive nations have strengthened their control over resource pricing and trade flows through the depth of financial markets and logistics networks.

#### 3.2.2 Core trade countries’ trade flow paths.

**Fig 9:**
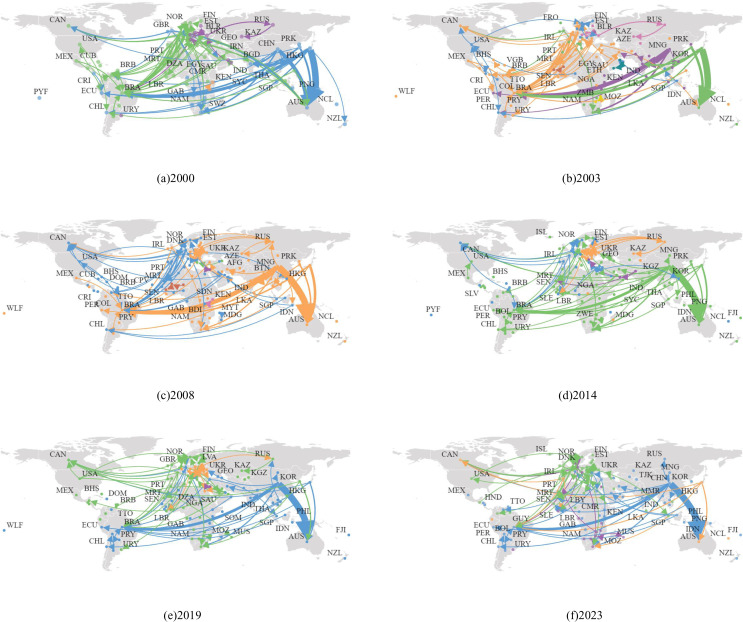
Major trade flows of iron ore at key time points. (Node: represent countries, arrows indicate the direction from importers to exporters, the thickness of the connecting lines represents the weight of the edges, i.e., the trade volume. The color of the edge is determined by the source node. Map base data source: Gapminder, https://graphica.app/).

Fig 9 visually illustrates the evolution of global iron ore trade flows from 2000 to 2023. The core trade network has maintained a high degree of stability, with Australia and Brazil continuing to dominate the export side as key resource-supplying countries, while Europe and Asia form the primary import regions. Notably, the Asian trade landscape has undergone significant differentiation. Japan’s position as the largest importer during the 2000–2003 period was replaced by China after 2003, a shift directly linked to China’s growth in steel production capacity. Europe exhibits a distributed pattern characterized by diversified small-scale imports and multilateral small-scale exports, while China has formed a single-dependent model of concentrated large-scale imports and limited exports. The majority of its imports have long been concentrated in Australia and Brazil, highlighting strategic risks in resource acquisition. The globalization process has profoundly restructured the spatial structure of the global iron ore trade network, exhibiting an evolutionary pattern of core stability and peripheral expansion. The traditional Pacific trade routes centered on Australia and Brazil remain dominant, but the active participation of developing countries such as India and South Africa has significantly expanded the coverage of the trade network, forming a multi-directional radiation pattern. The constraining effect of geographical distance on trade flows has weakened, reflecting the breakthrough of traditional locational constraints through advancements in logistics technology and supply chain management optimization. Specifically, this is manifested in: new land transport corridors expanding intra-Eurasian trade; the number of trade routes and the scope of participating countries continuing to expand, driving improvements in global connectivity; trade relations between developed countries remaining relatively stable, while the trade vitality of emerging markets has strengthened, with growth rates significantly higher than those of developed countries. This evolution of a trade pattern characterized by both concentration and dispersion reflects both the trend toward diversified resource supply and the economic logic of major importing countries enhancing their risk-resilience through supply chain diversification strategies.

### 3.3 Analysis of network community division and its evolution characteristics

This section analyzes the community division and evolution of the GIOTN through visualisation of community structure, focusing on the division outcomes, changes in the number of key members, and the overall evolutionary process.

#### 3.3.1 Network community division.

Community analysis is a key method in complex network research for identifying clusters of economies with strong internal connections. This study applies a modularity maximization algorithm to systematically partition the community structure of the GIOTN over the period 2000–2023.

Table 2 shows that the modularity index of the GIOTN underwent phased changes from 2000 to 2023: it rose gradually from 0.30 in 2000–2006 to 0.49, indicating strengthened intra‑community ties and weakened inter‑community links, with early signs of regional clustering; declined progressively to 0.06 in 2006–2014, reflecting enhanced inter‑community connectivity and greater trade network integration; peaked at 0.50 in 2015, when the network split into multiple highly autonomous trade communities, underscoring a marked trend toward regional cooperation; fell back to 0.08 in 2015–2020 as inter‑community links intensified and the network reintegrated, demonstrating the resilience of globalization; and rose again in 2021–2023, signaling stronger intra‑regional cluster connectivity in the post‑pandemic era and the reemergence of regional cooperation as a dominant feature. These phased shifts reveal alternating adjustments in the strength of intra‑ and inter‑community ties.

**Table 2:** Modularity of community division from 2000 to 2020.

Year	Modularity	Year	Modularity
2000	0.3	2012	0.07
2001	0.17	2013	0.14
2002	0.3	2014	0.06
2003	0.28	2015	0.5
2004	0.39	2016	0.07
2005	0.36	2017	0.07
2006	0.49	2018	0.15
2007	0.19	2019	0.08
2008	0.16	2020	0.08
2009	0.07	2021	0.35
2010	0.07	2022	0.06
2011	0.08	2023	0.28

**Fig 10:**
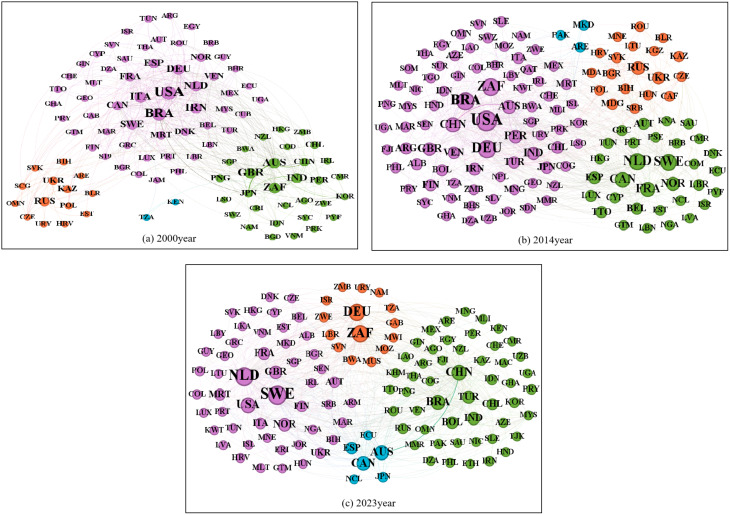
Community division of the GIOTN at key time points. (Note: Node size represents the total weight of all connected edges of a node. Edge weight represents trade volume, and line thickness represents trade value. The same color represents the same trade association, while different colors represent different trade associations.).

Quantitative analysis of community size and node weight (Fig 10, S3 Appendix) reveals the evolution of the GIOTN community structure from 2000 to 2023. In 2000, the network exhibited a dual structure dominated by U.S.–Brazil and Australia–U.K. trade relations (Fig 10a). By 2003, it had differentiated into five relatively independent blocs: Australia, Brazil, China, the U.S., and Russia (Fig 1a in S3 Appendix). After the 2008 financial crisis, the network polarized into two camps (Fig 1b in S3 Appendix): one centered on China, Brazil, Russia, Australia, and South Korea, the other comprising Europe, the U.S., and Canada. In 2014, the network further segmented into three distinctive groups (Fig 10d): purple (China, U.S., Brazil, Australia, South Africa), green (EU countries), and orange (Russia, Ukraine) (Fig 10b). In 2019, external factors reduced bilateral U.S.–China trade flows and weakened Brazil’s export capacity (Fig 1c in S3 Appendix). By 2023, the network formed four trade clusters (Fig 10c), dominated by two major ones: purple (Netherlands, Switzerland, U.S., France, U.K.) and green (China, Brazil, India, Turkey).

#### 3.3.2 Analysis of network community evolution characteristics.

**Fig 11:**
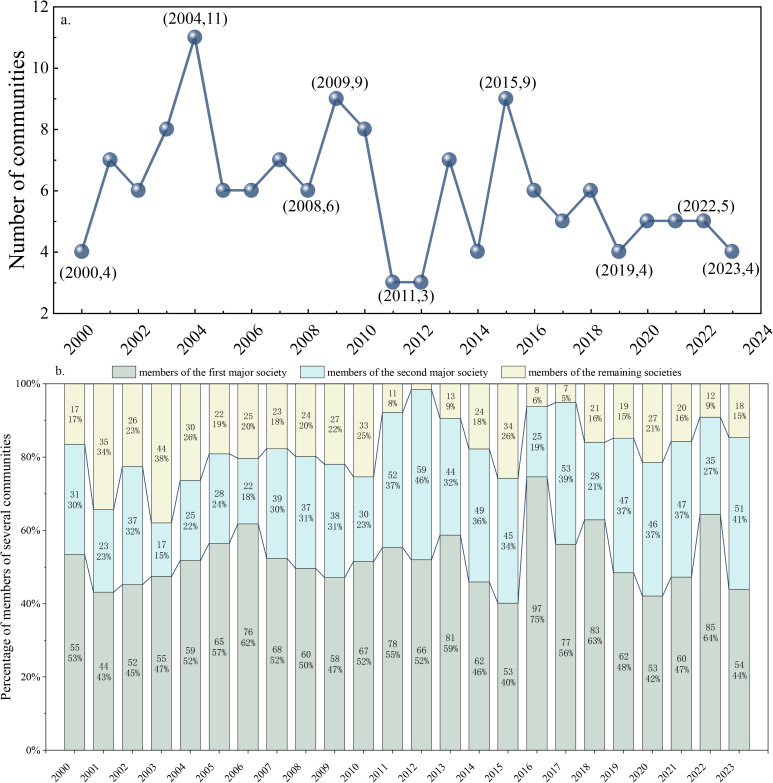
Changes in GIOTN community numbers and proportions of major community members. (Note: Figure a shows the changes in the number of GIOTN communities from 2000 to 2023, where the x-axis represents the year and the y-axis represents the number of communities in the corresponding year; Figure b shows the changes in the total number of members of major GIOTN communities from 2000 to 2023. The numbers in the bar chart represent the number of members of major communities, while the percentages indicate the proportion of major community members relative to the total number of community members. The total number of community members is calculated by summing the total number of members from all major communities.).

Fig 11a reveals three characteristics in the evolution of community numbers in the GIOTN. First, resource consolidation reduces community count: trade resources concentrate among fewer actors under economic and diplomatic forces, prompting mergers of smaller communities, while advances in international logistics weaken trade boundaries, leading to a continued decline in community numbers. Second, overlapping economic cycles and geopolitics induce periodic fluctuations: buoyant phases boost regional activity and spawn smaller communities, whereas downturns drive consolidation; resource competition among major powers reshapes alliances via shifting international relations, also causing cyclical variations. Third, rising market maturity enhances stability: the concentration of economic strength in leading countries, together with standardized pricing mechanisms, shifts the market from fragmentation to centralization, ultimately yielding a dynamic equilibrium in community numbers.

Fig 11b further illustrates the dynamic evolution of market power structure: from 2000 to 2006, the share of the largest community rose from 53% to 62%, while that of the second largest fell from 31% to 18%, widening the gap and forming a unipolar monopoly; from 2007 to 2015, the second largest community’s share rebounded, moving the market toward a duopoly equilibrium; in 2016, the largest community’s share peaked at 75%, after which its dominance gradually weakened amid accelerating globalization, while the second largest’s share continued to rise; by 2023, the shares of the largest and second largest communities stood at 44% and 41%, respectively, near parity, marking a shift from absolute monopoly to relative balance in the global iron ore trade market structure.

## 4. Research conclusions and policy recommendations

### 4.1 Research conclusions

Based on a global iron ore trade model constructed using complex network theory for the period 2000–2023, this study reveals three core conclusions regarding the evolution of global iron ore trade patterns. The global iron ore trade power structure is undergoing a strategic shift from resource-driven to capital-driven dominance. The global iron ore trade exhibits significant spatial mismatches between supply and demand. Resource-rich countries such as Australia and Brazil form a supply alliance with capital and technology-driven economies like Europe and North America, while China, as the absolute demand center, dominates the import market. This asymmetric structure has led to a shift in market control from traditional resource ownership to financial capital dominance.

The global iron ore trade flow pattern is undergoing a restructuring trend characterized by both diversification and regionalization. Developed countries such as Europe and North America, leveraging their strong mining capital, have established a flexible trade model featuring “multiple sources and small-scale imports, as well as exports to multiple countries in small volumes,” thereby forming a diversified supply chain system; In contrast, China has developed a typical pattern of “large-scale concentrated imports and small-scale exports.” Geopolitical shifts and the restructuring of international relations have accelerated the integration of trade communities, with a noticeable reduction in the number of communities and a trend toward regional consolidation, forming a stable landscape dominated by Europe, China, and Australia. China is seeking strategic breakthroughs by developing new supply channels in Africa, India, and other emerging markets, gradually reducing its reliance on traditional supply networks, and driving the evolution of the global iron ore trade landscape from an Australia-Brazil-dominated structure toward a more multipolar direction.

The global iron ore trade network remains highly active, yet the competitive landscape has become increasingly concentrated, with a few economies controlling the bulk of trade shares. Resource‑rich countries hold stable advantages on the supply side, while demand exhibits a divergent pattern. As mining ownership is largely concentrated in selected developed countries, resource‑rich nations can dominate trade flows but possess limited influence over pricing and rule‑making, resulting in a power asymmetry that favors capital holders over resource controllers. Against this backdrop, emerging economies are gradually reshaping the global trade pattern and enhancing their voice by strengthening demand‑side influence and deepening regional cooperation. Based on network topology and nodal power analysis, this study constructs a theoretical framework for understanding the dynamic evolution of the global iron ore resource trade system, offering a reference for formulating strategic resource governance policies.

### 4.2 Policy recommendations

Amid global low carbon transition and rising geopolitical uncertainty, iron ore, a key strategic input for the steel industry, has made supply security a major concern for importing countries. Based on analysis of global trade patterns and risk profiles, importers should develop a resource security framework aligned with current conditions and risks across four dimensions: geopolitical resilience, regional coordination, supply chain control, and green transition. Recommendations are outlined below.

In international resource governance, competition for rule‑making authority shapes the structure of trade communities, where multipolar community trends intertwine with capital‑dominated pricing. Importing countries should deepen cooperation with the EU in resource governance to address geopolitical challenges. The EU enhances its strategic autonomy and leads rule‑shaping through the Critical Raw Materials Act (CRMA) and transnational mechanisms. Importing countries ought to prioritize participation in the EU’s Critical Raw Materials Partnership to stabilize supply and reduce disruption risks [42]; engage in multilateral resource dialogue mechanisms to strengthen supply chain bargaining power through international rule‑making; and join green technology cooperation platforms to acquire advanced technologies that improve resource efficiency and supply chain resilience.

The global iron ore trade is shifting from a dual‑core community dominated by Australia and Brazil toward a multipolar structure; yet structural dependence on a few countries remains largely unchanged. Importing nations should promote diversification of import sources and establish regional strategic cooperation mechanisms. On one hand, they should expand collaboration with emerging resource‑rich countries such as South Africa, Ukraine, Malaysia, and Belt and Road partner states, securing a stable and diversified supply network through long‑term supply agreements, joint development of overseas mines, and investment in ports and shipping routes. On the other hand, they may draw on the EU’s CRMA [43], which caps the share of imports from any single third country at 65% and strengthens risk assessment [44], and leverage regional frameworks such as RCEP and ASEAN+3 to develop joint iron ore procurement platforms, thereby enhancing collective bargaining power and strengthening the long‑term resilience and risk resistance of supply chains.

As trade communities shift from centralized to multipolar structures, cross‑community transmission of logistics and production capacity volatility intensifies, and importing countries need to strengthen mineral resource reserve mechanisms to mitigate potential supply shocks. First, they should monitor dynamically the evolution of mine production capacity and reserves, define reserve targets, scale, and activation mechanisms, and use them as buffers during supply disruptions, price fluctuations, or geopolitical conflicts. Second, they should accelerate development of multimodal transport corridors and deepen infrastructure connectivity with resource‑rich countries to enhance supply chain emergency response and shock resistance. Third, learning from the EU CRMA approach that emphasizes supply chain monitoring, identification of logistical bottlenecks, and establishment of emergency reserves to ensure availability of critical raw materials during crises, they should integrate logistics assurance into the overall design of resource security strategy, take into account iron ore ownership structures and the influence of capital sources on pricing power and supply security, and assess and diversify capital dependency risks in overseas investments.

The global iron ore trade is characterized by resource-rich countries controlling trade flows while capital-rich nations dominate pricing power. Importing nations must adopt multi-dimensional strategies to enhance resource security and supply chain autonomy. First, encourage financial capital and steel enterprises to collaborate in “going global” to expand resource acquisition channels. Second, promote partnerships between steel companies and financial institutions to invest in resource-rich yet politically stable projects in regions like Africa and Central Asia, prioritizing assets with diversified capital structures to avoid reliance on single sources of capital. Third, support the establishment of non-dollar-denominated iron ore spot or futures trading platforms to diversify pricing systems, reduce dependence on the dollar system, and enhance bargaining power in transactions. Fourth, drawing on the European Union’s practice of strengthening economic ties with resource-rich nations through investments in critical infrastructure to reduce reliance on single supply chains and monetary systems, incorporate such geo-economic thinking into building China’s own resource control network to enhance supply chain resilience and autonomy.

Dependence on high‑grade ore increases supply vulnerability, and importing countries should establish a green steel industry collaboration system covering the entire chain of technological transformation. First, exploration and mining should focus on hybrid extraction technologies; material recycling should promote “scrap steel substitution for ore,” improve recovery systems, and raise recovery rates [45]; steel processing and substitution should develop alternative technologies such as alloy materials to alleviate demand‑side reliance on iron ore sources. Second, they should monitor the evolution of international regulations such as the EU Carbon Border Adjustment Mechanism (CBAM), guard against trade barriers arising from incompatible low‑carbon standards, introduce advanced resource‑utilization technologies through international cooperation, and enhance resource efficiency and supply chain resilience.

## Supporting information

S1 Appendix Changes in the share of iron ore trade export and import among major participating countries. (DOCX)

S2 Appendix Detailed introduction to the formula for iron ore trade network topology indicators. (DOCX)

S3 Appendix Community division of the GIOTN at key time points. (DOCX)
