# Supplementary material for: Analysis of the spatio-temporal evolution of iron ore trade from a geopolitical perspective: A complex network model
Source: PLoS One. 2026 Mar 24;21(3):e0345177. doi: 10.1371/journal.pone.0345177 (PMC13012449; doi:10.1371/journal.pone.0345177)
Supplement: S1 Appendix — (DOCX) [file pone.0345177.s001.docx]

**Supple mentary file 1**

Supplement data based on the timing of changes in the share of iron ore trade imports and exports between major reference countries.


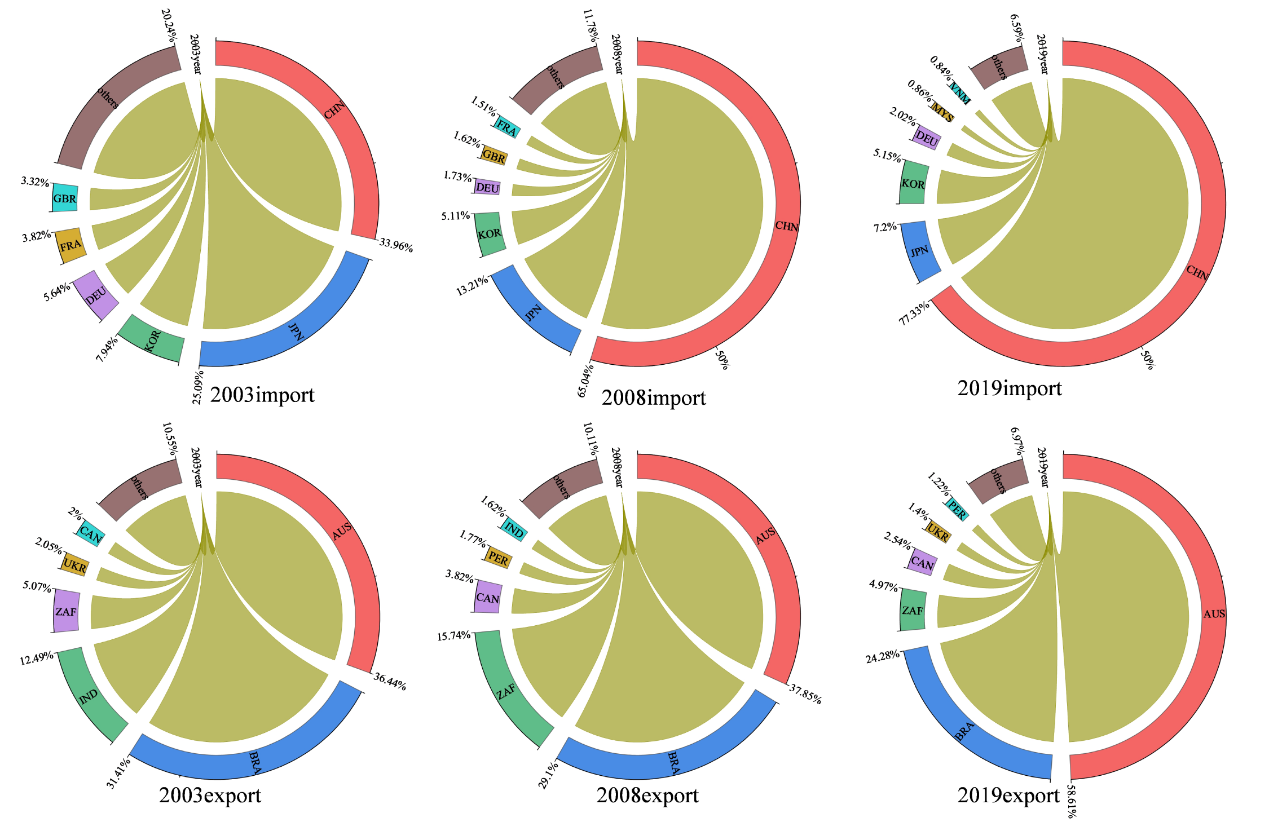


**S1 Fig.** **Changes in the Share of Iron Ore Trade Export and Import Among Major Participating Countries.**
