# Supplementary material for: Analysis of the spatio-temporal evolution of iron ore trade from a geopolitical perspective: A complex network model
Source: PLoS One. 2026 Mar 24;21(3):e0345177. doi: 10.1371/journal.pone.0345177 (PMC13012449; doi:10.1371/journal.pone.0345177)
Supplement: S2 Appendix — (DOCX) [file pone.0345177.s002.docx]

**Supple mentary file 2**

The following is a detailed introduction to the formula for iron ore trade network topology indicators.

### 1.Descriptive indicators of network structure

(1)Degree and degree distribution

The formula for calculating the average degree is as follows: $k_{i}$is the degree of node $i$; $N$is the set of nodes in the network. $i$ and $j$ are two distinct nodes i

(1)

The formula for calculating the weighted degree is as follows[1]: where $\omega_{ij}$ represents the weight of the edge between node $i$ and node $j$

$w_{i}^{in}=\sum_{j=1}^{N} w_{ji}$ (2)

$w_{i}^{out}=\sum_{j=1}^{N} w_{ij}$ (3)

The degree distribution represents the proportion of nodes with degree $k$ in the entire network. The formulais as follows[2]: where $N_{k}$ is the number of nodes with degree$k$.

$p_{k}=\frac{N_{k}}{N}$ (4)

The power-law distribution is given by the following formula[3]: where $\gamma$ is the power-law exponent, which is a constant.

$p_{k}=ck^{-\gamma}$ (5)

(2)Network density

The formula for calculating network density $\rho$ is as follows[4]: where $E$ represents the actual number of connections in the network.

$\rho=\frac{E}{N(N-1)}$ (6)

(3)Average path length

The formula for Average Path Length is as follows: where $d_{ij}$ represents the length of the shortest path between nodes $i$ and $j$

$L=\frac{2}{N(N+1)}\sum_{i\neq j} d_{ij}$ (7)

### 2.Descriptive node importance index

(1)Betweenness centrality

The formula for calculating betweenness centrality is as follows: where $\sigma_{ij}$ is the total number of shortest paths between nodes $i$ and $j$, $\sigma_{ij}(v)$ is the number of shortest paths that pass through node $v$. Node $v$ is the node being examined.

$C_{B}(v)=\sum_{\begin{matrix} i\neq j\neq v \\ i,j\in N \end{matrix}}\frac{\sigma_{ij}(v)}{\sigma_{ij}}$ (8)

(2)Closeness centrality

The formula for calculating closeness centrality is as follows[5]: where $d_{ij}$ represents the shortest path length between node $i$ and node $j$.

$C_{C}(v)=\frac{N-1}{\sum_{j=1}^{N} d_{ij}}$ (9)

(3)Eigenvector centrality

The formula for calculating eigenvector centrality is as follows: where $\lambda$ is a proportionality constant, represents the set of nodes adjacent to node $i$, and $\omega_{ij}$ represents the connection weight between node $i$ and node $j$.

$C_{E}(v)=\frac{1}{\lambda}\sum_{j\in M(i)} \omega_{ij}C_{E}(v)$ (10)

### 3. Network community partition

Modularity is a commonly used method to measure the strength of a network's community structure. The formula is as follows:

$Q=\frac{1}{2m}\sum_{i,j} \left[\omega_{i,j}-\frac{A_{i}A_{j}}{2m}\right]\delta(c_{i},c_{j})$ (11)

where $m=\frac{1}{2}\sum_{i,j} \omega_{ij}$ represents the total weight of edges in the network, $A_{i}=\sum_{j} \omega_{ij}$represents the sum of the weights of edges connected to node $i$, and $\delta(c_{i},c_{j})$ is used to determine whether node $i$and node $j$ belong to the same community. If$c_{i}=c_{j}$,then$\delta=1$,otherwise$\delta=0$.

**References**

1. Garlaschelli D, Loffredo MI. Structure and evolution of the world trade network. Phys -Stat Mech ITS Appl. 2005 Sep 1;355(1):138–44.
2. Chen B, Li JS, Wu XF, Han MY, Zeng L, Li Z, et al. Global energy flows embodied in international trade: A combination of environmentally extended input-output analysis and complex network analysis. Appl ENERGY. 2018 Jan 15;210:98–107.
3. Barabási AL, Albert R. Emergence of scaling in random networks. In: The Structure and Dynamics of Networks. 2011. p. 349–52.
4. Hu X, Wang C, Lim MK, Koh SCL. Characteristics and community evolution patterns of the international scrap metal trade. J Clean Prod. 2020 Jan 10;243:118576.
5. Bavelas A. Communication Patterns in Task-Oriented Groups. J Acoust Soc Am. 1950;22(6):725–30.
