# Supplementary material for: Analysis of the spatio-temporal evolution of iron ore trade from a geopolitical perspective: A complex network model
Source: PLoS One. 2026 Mar 24;21(3):e0345177. doi: 10.1371/journal.pone.0345177 (PMC13012449; doi:10.1371/journal.pone.0345177)
Supplement: S3 Appendix — (DOCX) [file pone.0345177.s003.docx]

**Supple mentary file 3**

Supplementary data on community classification by GIOTN at key time points.


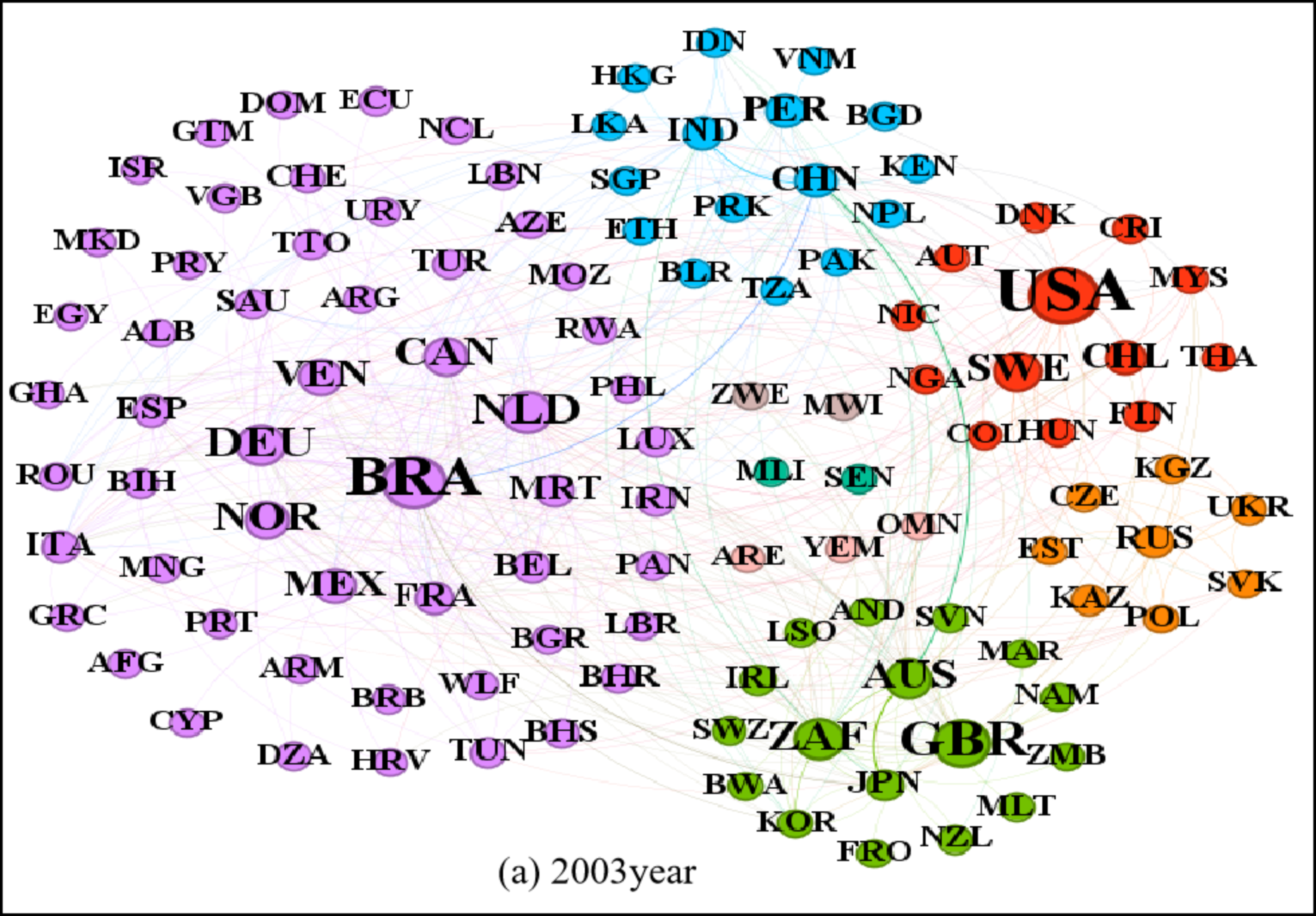


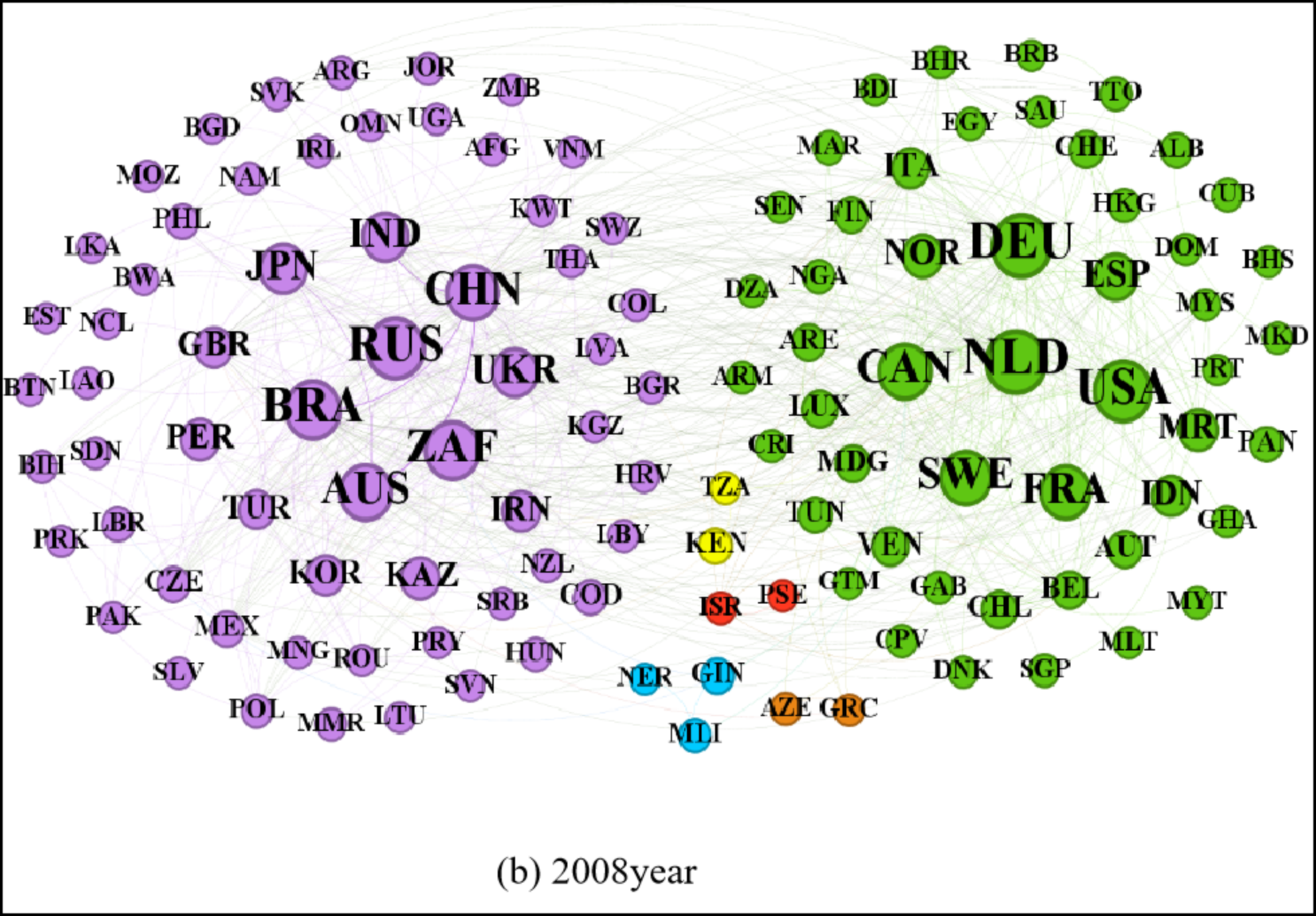


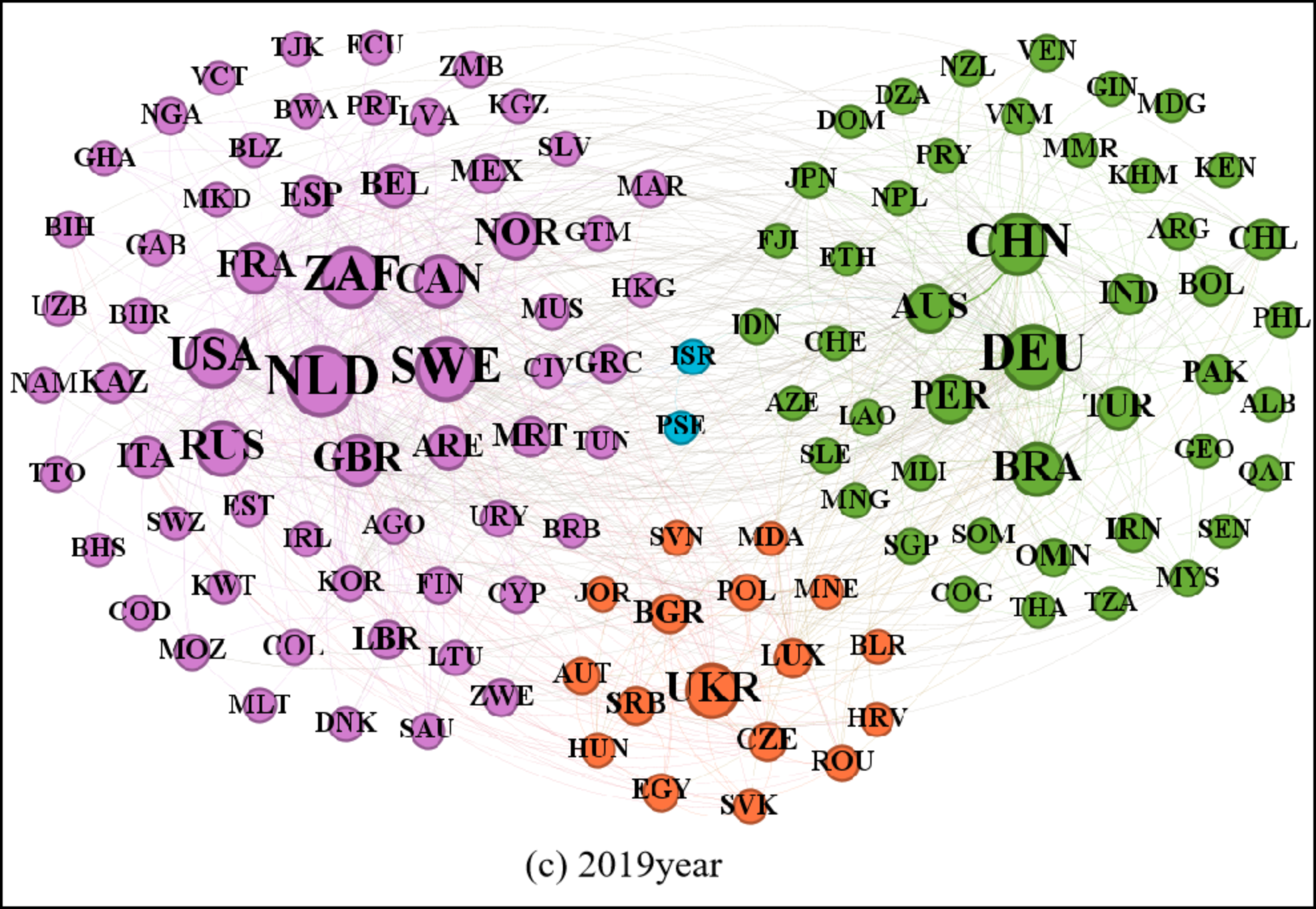


**S3 Fig1. Community Division of the GIOTN at Key Time Points .** (Note: Node size represents the total weight of all connected edges of a node. Edge weight represents trade volume, and line thickness represents trade value. The same color represents the same trade association, while different colors represent different trade associations.)
